# An evaluation of oligonucleotide-based therapeutic strategies for polyQ diseases

**DOI:** 10.1186/1471-2199-13-6

**Published:** 2012-03-07

**Authors:** Agnieszka Fiszer, Marta Olejniczak, Pawel M Switonski, Joanna P Wroblewska, Joanna Wisniewska-Kruk, Agnieszka Mykowska, Wlodzimierz J Krzyzosiak

**Affiliations:** 1 Laboratory of Molecular Biomedicine, Institute of Bioorganic Chemistry, Polish Academy of Sciences, Noskowskiego 12/14, 61-704 Poznan, Poland

**Keywords:** Triplet repeats, Polyglutamine diseases, siRNA, Antisense oligonucleotides, SNP targeting, CAG repeat targeting

## Abstract

**Background:**

RNA interference (RNAi) and antisense strategies provide experimental therapeutic agents for numerous diseases, including polyglutamine (polyQ) disorders caused by CAG repeat expansion. We compared the potential of different oligonucleotide-based strategies for silencing the genes responsible for several polyQ diseases, including Huntington's disease and two spinocerebellar ataxias, type 1 and type 3. The strategies included nonallele-selective gene silencing, gene replacement, allele-selective SNP targeting and CAG repeat targeting.

**Results:**

Using the patient-derived cell culture models of polyQ diseases, we tested various siRNAs, and antisense reagents and assessed their silencing efficiency and allele selectivity. We showed considerable allele discrimination by several SNP targeting siRNAs based on a weak G-G or G-U pairing with normal allele and strong G-C pairing with mutant allele at the site of RISC-induced cleavage. Among the CAG repeat targeting reagents the strongest allele discrimination is achieved by miRNA-like functioning reagents that bind to their targets and inhibit their translation without substantial target cleavage. Also, morpholino analog performs well in mutant and normal allele discrimination but its efficient delivery to cells at low effective concentration still remains a challenge.

**Conclusions:**

Using three cellular models of polyQ diseases and the same experimental setup we directly compared the performance of different oligonucleotide-based treatment strategies that are currently under development. Based on the results obtained by us and others we discussed the advantages and drawbacks of these strategies considering them from several different perspectives. The strategy aimed at nonallele-selective inhibiting of causative gene expression by targeting specific sequence of the implicated gene is the easiest to implement but relevant benefits are still uncertain. The gene replacement strategy that combines the nonallele-selective gene silencing with the expression of the exogenous normal allele is a logical extension of the former and it deserves to be explored further. Both allele-selective RNAi approaches challenge cellular RNA interference machinery to show its ability to discriminate between similar sequences differing in either single base substitutions or repeated sequence length. Although both approaches perform well in allele discrimination most of our efforts are focused on repeat targeting due to its potentially higher universality.

## Background

PolyQ diseases, which are caused by the expansion of CAG repeats in the translated sequences of different genes, include Huntington's disease (HD), several spinocerebellar ataxias (SCAs), dentatorubral pallidoluysian atrophy (DRPLA) and spinal bulbar muscular atrophy (SBMA) [1]. The expansion of a repeated sequence in DNA results in the formation of mutant transcripts and the translation of mutant proteins containing polyQ tracts, which leads to the degeneration of neurons within specific regions of the brain and spinal cord in some cases [2,3]. The pathogenesis of polyQ diseases is triggered by a single mutant allele of the implicated gene acting in a dominant "gain-of-function" fashion [4]. Mutant protein toxicity is considered to be main pathogenic factor, but mutant transcripts may also contribute to the pathogenesis [5-8].

RNAi is a powerful tool for specific silencing of disease-causing genes [9]. The introduction of RNAi reagents, mainly short interfering RNAs (siRNAs) or short hairpin RNAs (shRNAs), into cells leads to the sequence-specific cleavage of transcripts containing the complementary sequences by RNA-induced silencing complexes (RISCs) [10,11].

The nonallele-specific silencing of genes causing HD and SCA3 was shown to be beneficial in rodent models of these diseases, suggesting that a partial loss of normal protein function can be tolerated [12-15]. Other studies suggest that for some polyQ disorders, the wild-type normal protein expression is required for cellular function, indicating that allele-specific therapeutic strategies would be more appropriate [16-18]. Because the effect of the efficient silencing of both alleles of a specific gene in human cells is difficult to predict, the allele-specific inhibition of the mutant gene expression is considered a safer and more promising strategy for causative therapies. For polyQ diseases, the transcript regions that distinguish the alleles can be targeted by RNAi reagents: single-nucleotide polymorphisms (SNPs) linked to repeat expansions or the repeat region itself. SNP targeting has been previously described for HD [19-23], SCA3 [24-26] and SCA7 [27]. The low frequency of suitable allele-distinguishing SNPs in the human population limits this strategy; therefore, prospective treatments must be tailored to individual patients. Several authors have shown that targeting several selected SNPs can be applied to the majority of HD patients [20,28,29]. Targeting the repeat region by RNAi reagents could offer a therapeutic approach for all polyQ diseases, but CAG-repeat-targeting siRNAs have insufficient allele-discriminating properties [26,30]. Allele-specific inhibition of the proteins implicated in HD and SCA3 has been demonstrated with repeat-targeting antisense PNA (peptide nucleic acid) and LNA (locked nucleic acid) oligomers [31,32] and miRNA (microRNA)-like RNA duplexes [30,33].

Determining the most effective and safe potential treatment of each specific polyQ disease is difficult. In this study, we tested several oligonucleotide-based strategies for selected CAG-repeat-containing genes. We discuss the advantages and disadvantages of the different approaches. We targeted the *HTT, ATXN3 *and *ATXN1 *genes, which are implicated in HD, SCA3 and SCA1, respectively. In our experimental setup, we used cell culture models to evaluate the effect of the different strategies on endogenously expressed targets instead of an artificial exogene targeting system. We discuss the potential of each approach for use in further testing.

## Results and discussion

### Nonallele-selective silencing by targeting specific *HTT *sequences

Gene silencing without discrimination of alleles can be achieved by RNAi-based targeting of a specific sequence within a gene. We analyzed *HTT *silencing by typical RNAi reagents in a cellular model of HD. We used a fibroblast cell line (GM04281, 17/68 CAG in *HTT *mRNA) to compare the silencing efficiencies of different *HTT*-specific siRNAs (Additional file 1: Figure S1A). First, siRNA HD1 was transfected at 50 nM. The *HTT *mRNA and protein levels were assessed using RT-PCR and western blot, respectively, at the indicated time points (Additional file 1: Figure S1B, C). A substantial decrease in *HTT *mRNA to ~25% of the control level was observed 24 h after transfection. After 72 h, the level of transcript began to slowly increase (Additional file 1: Figure S1B). A strong reduction in the level of HTT protein occurred 48 h after transfection, and the levels remained low for at least the next 6 days (Additional file 1: Figure S1C). As expected, no allele discrimination of HTT silencing by a specific siRNA was observed.

All three *HTT*-specific siRNAs (HD1, HD2 and HD3) contained 21-nt strands, and their targets were located within the *HTT *mRNA portion encoded by exon 1. The specific siRNAs tested showed similar silencing activities on the *HTT *transcripts (Additional file 1: Figure S1D). The efficiency of the inhibition of the mutant HTT protein was tested for HD1 siRNA at different concentrations (Additional file 1: Figure S1E). HD1 induced an approximately 50% reduction of the HTT protein level at 2 nM and an approximately 75% reduction at 50 nM.

As shown previously by others and in our experiments with the three siRNAs, effective *HTT *silencing can be easily achieved in cell culture models and in mouse models of HD [34,35]. This kind of reagent can be easily converted into shRNA for the induction of long-term silencing effects [36,37].

Interestingly, the nonallele-specific silencing of *HTT *was shown to provide several benefits in rodent models of HD [13,14] and in nonhuman primates [38]. The authors reported improved motor coordination and survival in HD mice after silencing of both *htt *alleles and well-tolerated reduced levels of endogenous wild-type *Htt *within the striatum in the rhesus macaque.

### The nonallele-selective silencing of *ATXN3 *with the replacement of the normal allele

The effective silencing of both alleles of a gene implicated in a disease can be accompanied by the exogenous expression of the normal allele to preserve normal protein function. This so-called replacement strategy was previously tested for SCA6 [39,40], ALS (amyotrophic lateral sclerosis) [41] and RP (retinitis pigmentosa) [42,43].

**Figure 1:**
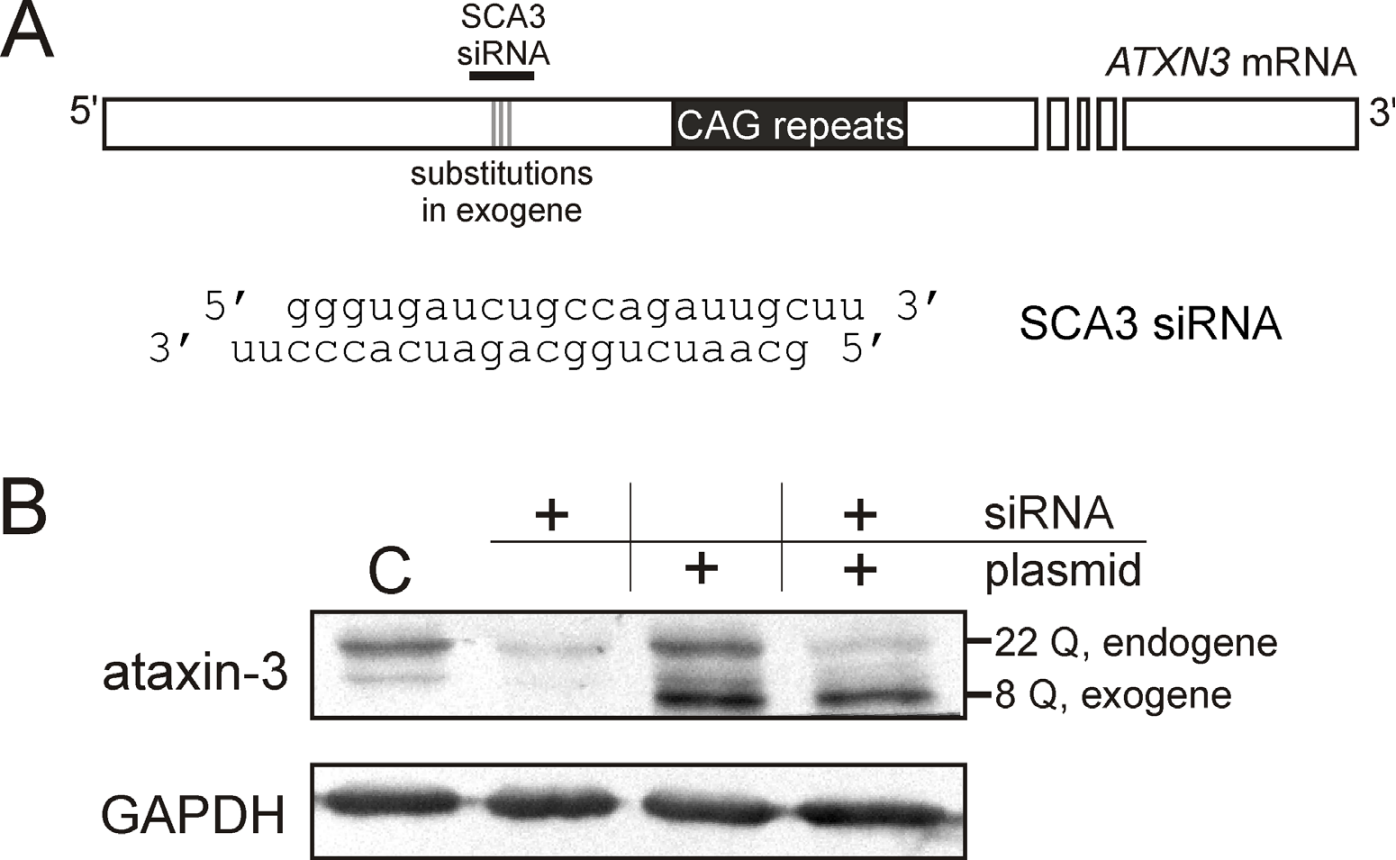
**The replacement strategy tested for *ATXN3***. **(A) **The target site and nucleotide sequence of siRNA directed at the *ATXN3 *transcript. **(B) **The western blot analysis of the ataxin-3 levels in the HeLa cells at 72 h after transfection with 100 nM siRNA SCA3 and/or ATXN3-8Q plasmid. C - the reference line shows the *ATXN3 *expression level in the cells transfected with control siRNA. The GAPDH protein level was used as a loading control.

We chose the *ATXN3 *transcript for the evaluation of this strategy (Figure 1A). First, we tested specific siRNA for efficient nonallele-selective silencing of *ATXN3 *expression. Then we cloned the cDNA of *ATXN3 *containing 8 CAG repeats and introduced specific mutations at the siRNA target sequence to make the exogene transcript resistant to RNAi. The substitution positions in the target sequence were 7, 10 and 16 nt from the 5' end of the antisense siRNA strand. These mutations did not change the amino acid sequence of ataxin-3.

The testing of the replacement strategy for *ATXN3 *was performed in HeLa cells due to the significant toxicity induced by the plasmid encoding exogenous ataxin-3 in the SCA3 fibroblasts. The HeLa cells were transfected with *ATXN3*-specific siRNA and/or ATXN3 plasmid (ATXN3-8Q), and the ataxin-3 level was monitored by western blotting. Co-transfection with siRNA and the ATXN3 plasmid resulted in a significant reduction of endogenous ataxin-3 (22 Q) and the simultaneous expression of exogenous ataxin-3 (8 Q) at a level comparable to that of the endogene (Figure 1B). This effect obtained in the HeLa cell model system represents anticipated results of the replacement strategy, indicating that this approach is feasible.

The main advantage of the replacement strategy is that it provides the allele-specific effect of normal/mutant protein expression without the need to design allele-discriminating reagents. The main challenge of this approach is to obtain the appropriate level of exogene expression, which would correspond to the endogene expression level.

### The allele-selective silencing of polyQ genes by SNP-targeting

The SNP-targeting strategy is based on the ability of RISC to discriminate between two alleles of a gene that differ by a single nucleotide in the target sequence. When the siRNA guide strand is fully complementary to the mutant transcript, cleavage will occur. Otherwise, a single-nucleotide mismatch with the normal transcript can inhibit or prevent the cleavage reaction.

There are several guidelines for the design of SNP-targeting siRNAs based on the testing of RNAi reagents for specific genes and SNPs [20,21] and based on systematic large-scale studies [44,45]. These guidelines include the preferential positioning of mismatch (mainly central), the type of siRNA/mRNA mismatch (preferably purine-purine) and the possible introduction of additional mismatches.

Additionally, different guidelines for specific siRNA design can be used in the development of SNP-targeting siRNAs (reviewed in [46,47]). These guidelines include the manipulation of the siRNA length, the introduction of thermodynamic asymmetry into the siRNA duplex and the chemical modification of siRNA strands [48-53]. Different approaches can be tested to increase the efficiency and allele selectivity of the reagent. The efficiency is primarily enhanced by an increase in reagent stability or by making the antisense strand preferential for RISC loading.

Here, we show examples of the SNP-based approach for SCA3, HD and SCA1. The SCA1 model is evaluated in this strategy for the first time and the specific *HTT *SNP has not been investigated in previous studies.

First, we screened the *HTT, ATXN1 *and *ATXN3 *genes in the HD, SCA1 and SCA3 fibroblast cells, respectively, in search of suitable SNP variants that would differ between the normal and the mutant allele. Several cell lines were tested to find the heterozygous SNPs and the colocalization of SNP variants with either normal or mutant allele was demonstrated (Additional file 2: Supplementary Table 1 and Supplementary Methods).

#### Targeting ATXN3 SNP in SCA3 cells

**Figure 2:**
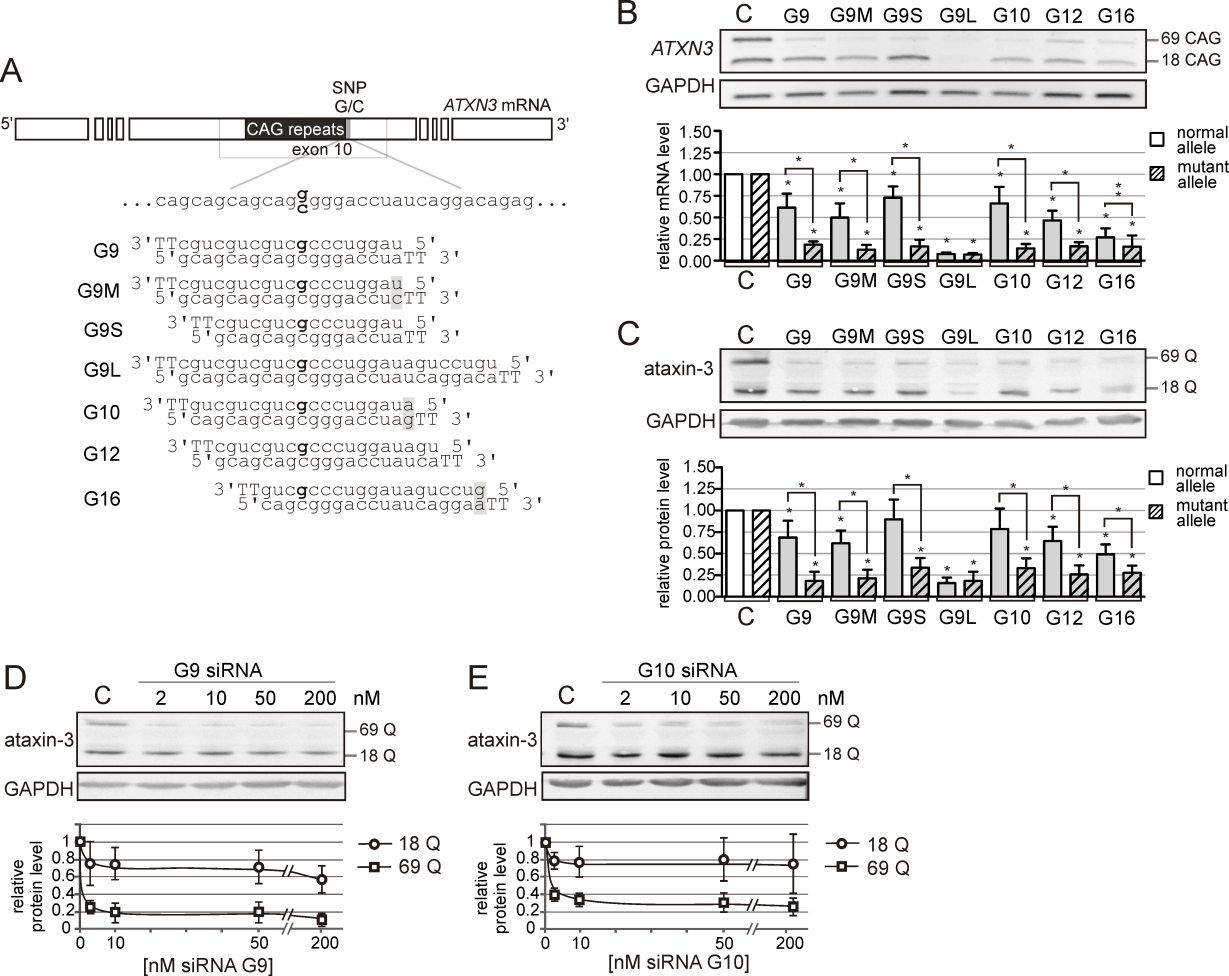
**The activity of siRNAs targeting SNP sites in the SCA3 model**. **(A) **The target sites and nucleotide sequences of siRNAs directed at rs12895357 SNP in the *ATXN3 *gene. The nucleotide in the antisense strand of siRNA that directly targets the SNP site is marked in bold. The mismatches introduced into siRNA duplex are in gray boxes. RNA is in lowercase; DNA is in uppercase. **(B) **The RT-PCR analysis of *ATXN3 *expression at the transcript level in the SCA3 fibroblasts (GM06153) at 72 h after transfection with 50 nM siRNAs. **(C) **The western blot analysis of the ataxin-3 levels for the experiment described in (B). **(D) **The western blot analysis of the ataxin-3 levels in the SCA3 fibroblasts at 72 h after transfection with 2, 10, 50 or 200 nM siRNA G9. **(E) **The western blot analysis as in (D) for siRNA G10. C - the reference bar indicates the *ATXN3 *expression level in the cells transfected with control siRNA. In the graphs, the signal intensities were normalized to GAPDH mRNA or protein levels and compared using a one-sample *t*-test. The error bars represent the standard deviations. The *P*-value is indicated by asterisks (* p < 0.001; ** 0.001 < p < 0.05).

For *ATXN3 *targeting, we selected the SNP that was used by others in exogenous models [24-26]. The selected SNP (rs12895357) is located at the 3' end of the repeat tract, and its G and C variants were shown to be associated with the normal and mutant alleles, respectively [25,54]. Worldwide, 70% of disease chromosomes carry the C variant which makes this SNP a promising target for this strategy [55]. Following the existing guidelines, we designed 7 siRNAs that are mismatched with the normal allele at positions 9 (G9), 10 (G10), 12 (G12) or 16 nt (G16) from the 5' end of the antisense siRNA strand (Figure 2A). We tested three variants of the G9 siRNA: one with a mismatch in the siRNA duplex (G9M); a second with shorter, 18-nt strands (G9S); and a third with longer, 27-nt strands (G9L). The siRNAs were transfected into the SCA3 cells (GM06153, 18/69 CAG in *ATXN3 *mRNA) at 50 nM. The *ATXN3 *mRNA and protein levels were assessed using RT-PCR and western blot, respectively (Figure 2B, C). All of the siRNAs were effective in mutant *ATXN3 *silencing, decreasing the mutant ataxin-3 levels to approximately 25% of the control level. Most of 7 siRNAs showed some allele-discriminating properties, with very high allele selectivity observed for G9, G9M, G9S and G10. The length-modified duplexes, G9S and G9L, showed decreased and increased silencing activity, respectively. This finding agrees with the effects typically observed after changes in siRNA length. The G9 and G10 siRNAs were then tested over a broader range of concentrations from 2 to 200 nM. Both siRNAs showed significant allele discrimination in ataxin-3 silencing for all of the concentrations tested (Figure 2C, D). The G9 siRNA showed higher efficiency in mutant allele silencing, with an ~80% reduction of mutant ataxin-3 even at 2 nM. For both siRNAs, normal ataxin-3 was retained to at least 60% of the control level even at 200 nM.

#### Targeting HTT SNPs in HD cells

To test the SNP-targeting strategy in the HD cellular model, we chose C/T SNP (rs1065745) located ~1 kb from the CAG repeat tract. Average heterozygo**s**ity of the SNP is 0,152 and the frequency of the minor allele T varies from 0% in European and Asian populations to 19,3% in Sub-Saharan African populations (NCBI dbSNP). We designed siRNAs with mismatches to the normal allele at positions 9 (G9), 10 (G10) or 16 nt (G16) (Additional file 3: Figure S2A). We also designed three variants of the G10 siRNA: two with additional mismatches in the target sequence (G10M1 and G10M2) and a duplex composed of 18-nt strands (G10S). The siRNAs were transfected into the HD cells (GM04208, 21/44 CAG in *HTT *mRNA) at 50 nM, and the *HTT *mRNA level was assessed using RT-PCR (Additional file 3: Figure S2B). Considerable allele selectivity was observed for the G16 siRNA. The other siRNAs showed either no allele-discriminating properties or no activity at all. We tested the *HTT *transcript and protein silencing by G16 over a broader range of concentrations from 2 to 200 nM (Additional file 3: Figure S2C, D). The G16 siRNA showed significant allele-discriminating properties at all of the concentrations tested, decreasing the normal mRNA to no more than 80% of the control level and silencing the mutant allele to ~40% of the control level. Next, we assessed protein inhibition by G16. Mutant allele expression was analyzed using a polyQ-specific antibody, and the whole-HTT level was analyzed using a huntingtin-specific antibody, which showed a decrease in the mutant protein level (Additional file 3: Figure S2D).

#### Targeting ATXN1 SNP in SCA1 cells

In the SCA1 model, we tested C/T SNP (rs179990) targeting. This SNP with an average heterozygosity of 0,41 is located ~250 nt from the CAG repeat tract. The minor allele T occurs with about 30% frequency in ESP cohort populations (the group includes some of the largest well-phenotyped populations in the United States) (NCBI dbSNP). We designed four siRNAs with mismatches relative to the normal allele at positions 9 (G9), 10 (G10), 11 (G11) or 16 nt (G16) (Additional file 4: Figure S3A). The siRNAs were transfected into SCA1 fibroblasts (GM06927, 29/43 CAG in *ATXN3 *mRNA) at 50 nM. The *ATXN1 *mRNA level was assessed using RT-PCR (Additional file 4: Figure S3B). All of the siRNAs showed relatively low efficiency in the silencing of the *ATXN1 *alleles with no more than a 60% reduction in the mRNA level. We only observed considerable allele selectivity for G10. We tested this siRNA over a broader range of concentrations (Additional file 4: Figure S3C). At 200 nM, there were ~20% and ~60% reductions in the normal and mutant allele levels, respectively. Significant allele discrimination in *ATXN1 *silencing was observed at all of the G10 concentrations tested.

#### The evaluation of SNP targeting

The SNP variants in the SCA3 model (18CAG/G and 69CAG/C) offer better properties for allele discrimination by siRNA because strong purine-purine mismatches (G-G, siRNA-normal allele target) can be introduced. For SNPs in the models of HD (21CAG/T and 44CAG/C) and SCA1 (29CAG/T and 43CAG/C), we were able to analyze allele selectivity based on the discrimination between G-U (weak) pairing on normal allele and G-C (strong) pairing on mutant allele. Nevertheless, for all of the models tested, the siRNAs showing considerable allele-discriminating properties were selected. The best performing reagents have a mismatch position located in the central part of the antisense strand (at position 9 or 10 counting from the 5'-end). For the HD model, the relevance of position 16 was confirmed [21].

A serious limitation of the SNP-targeting strategy is the restriction in the choice of the target sequence. Some regions containing SNPs may not be convenient targets for siRNAs, which may explain the low efficiency of the *ATXN1*-directed reagents in our experiments. We expected that for shorter siRNA duplexes, a 1-nt mismatch could result in stronger allele discrimination. However, for G9S and G10S, we only observed decreased activity with no improvement in the allele selectivity in *ATXN3 *and *HTT *silencing, respectively.

The existing guidelines for the design of SNP-targeting reagents are still not precise, and for most SNPs, a set of siRNAs must be tested to find the reagent that possesses the desired activity. Moreover, there may be problems with ensuring the long-term allele-selective silencing effects with shRNAs in the SNP-targeting strategy. shRNA is processed in cells by the RNase Dicer to generate siRNAs. It is difficult to predict the exact position of Dicer cleavage, and multiple cleavages often occur that differ by 1 or 2 nts [56,57]. For SNP-targeting siRNA, the effect of 1- or 2-nt differences in the mismatch position can result in a loss of allele-selective activity. Despite these limitations, allele-discriminating shRNAs based on SNP-targeting have been previously described [24].

### Repeat-region targeting for *HTT *silencing

Triplet repeat expansion can also be targeted by different oligonucleotide reagents. This straightforward strategy has some limitations resulting from the high frequency of relatively long CAG repeat tracts in normal transcripts [58]. Human genome encodes about 200 mRNAs harboring CAG repeats and about 100 mRNAs containing CUG repeats longer than 6 repeated units and transcripts having these sequences are potential unintended targets of regular CAG/CUG siRNAs (off-target effect). This effect is much less pronounced for modified siRNAs functioning like miRNAs and inhibiting translation rather than inducing mRNA cleavage. Therefore, gene selectivity and allele selectivity of silencing is more difficult to achieve in this case. Several RNAi reagents and chemically modified antisense reagents have been used for targeting of the repeat region in *AR, ATXN3, ATXN1 *and *HTT *[26,30,32,33,59,60].

#### Targeting CAG repeats by antisense reagents

**Figure 3:**
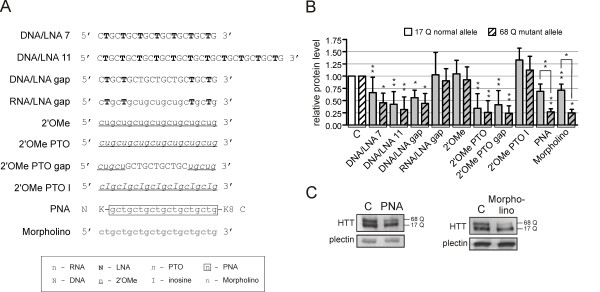
**Chemically modified oligomers as huntingtin inhibitors**. **(A) **The sequences of oligomers with indicated chemical modifications at particular positions. **(B) **The western blot analysis of huntingtin levels in the HD fibroblasts (GM04281) at 72 h after transfection with the indicated oligomers. **(C) **Representative results for the experiment described in (B) for allele-selective oligomers: PNA and morpholino. C - the reference bar indicates the *HTT *expression level in the cells transfected with control siRNA. In the graph, the signal intensities were normalized to the plectin protein level and compared using a one-sample *t*-test. The error bars represent standard deviations. The *P*-value is indicated by asterisks (* p < 0.001; ** 0.001 < p < 0.05).

Antisense oligonucleotides (ASOs) can be designed to silence the expression of polyQ-related genes according to different mechanisms, e.g., through translational blockade or RNaseH activation. There is also a large variety of chemical modifications that can be introduced into the reagent sequence. Here, we tested a set of CAG-repeat-targeting reagents, which included different mixmers containing LNA modifications. These reagents possessed pure DNA or RNA sequences in the central region (DNA/LNA gap and RNA/LNA gap) to activate specific RNA-RNA- or DNA-RNA-dependent cleavages of mRNA. We also designed oligomers containing 2'OMe (2'OMethylo), PTO (phosphothio) and morpholino modifications. All of the reagents are presented in Figure 3A. Our experiments also included the use of CAG/CUG siRNA (d7) [26,30], DNA/LNA 7 and PNA [32].

The oligomers were transfected into HD fibroblasts (GM04281, 17/68 CAG in *HTT *mRNA) at 50 nM, with the exception of PNA and the morpholino, which were delivered to cells at 2 μM and 20 μM, respectively. The huntingtin level was assessed by western blotting 72 h after transfection (Figure 3B). Most of the reagents showed silencing of both alleles of *HTT *or no activity at all. High allele selectivity was demonstrated for morpholino and was confirmed for PNA (Figure 3C). Both oligomers showed similar inhibitory properties with a decrease of mutant huntingtin to ~25% of the control level and normal allele silencing to no more than 70% of the control level.

In the cell culture experiments, PNAs and morpholinos are very difficult to deliver. Cell entry of these uncharged oligomers was facilitated by Endo-Porter delivery of the morpholinos and the addition of lysine residues to the PNAs. Very high concentrations were still required in the cell culture medium to observe any silencing effects. Nevertheless, the methods of delivery for such oligomers are under development [61], to take better advantage of their allele-selective activity.

#### Targeting CAG repeats by siRNA and miRNA-like reagents

**Figure 4:**
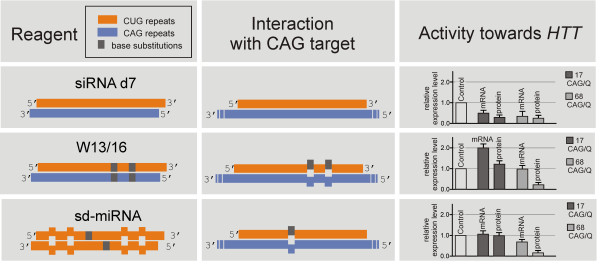
**A comparison of RNA duplexes targeting the CAG repeat tract in *HTT***. A schematic representation of the duplex sequences (left panel) and the interaction of the CUG strand with CAG tracts in the *HTT *transcript (middle panel). The right panel shows our accumulated results (published elsewhere) of duplex activity toward *HTT *expression at the mRNA and protein levels in the same experimental setup: 50 nM concentration of duplexes 72 h after the transfection of the HD cell line (GM04281).

High allele discrimination by repeat-region targeting cannot be achieved by typical RNAi reagents because siRNAs composed of CAG/CUG strands efficiently silence the expression of mutant and normal *HTT *and *ATXN3 *alleles [26,30,32]. We also characterized the activity of CAG/CUG siRNAs and showed that both strands of this duplex are active in the silencing of several other normal transcripts containing complementary repeats [30]. We and others have shown recently that the sequence modification of CAG/CUG siRNA results in allele-selective huntingtin inhibition [30,33]. These so-called miRNA-like reagents were designed to contain mismatches with the target sequence at specific positions, and they were found to be active at the level of the inhibition of mutant protein translation. In Figure 4, we summarize the tested approaches with RNA duplexes targeting CAG repeats in the *HTT *transcript, including our newly designed reagents, the so-called self-duplexing miRNAs (sd-miRNAs, Fiszer et al., manuscript submitted). The self-duplexing miRNAs are a new class of silencing reagents composed of CUG repeats that contain one or more U-base substitutions increasing the ability of oligoribonucleotide to form self-duplex structure. These guide-strand-only reagents show lower toxicity than regular siRNA and miRNA-like duplexes composed of both the CUG and CAG repeat strands.

#### Challenges connected with testing the repeat-targeting reagents

There are some difficulties involved with assessing the silencing activity of repeat-targeting reagents. We found that standard RT-PCR might be an unreliable method for the assessment of the potential degradation of transcripts targeted by chemically modified oligomers when the amplification includes the repeat region. The results, which indicate a preferential lowering of the mutant mRNA level by oligomers, may be misleading. This effect may stem from the inhibition of mutant mRNA reverse transcription by the excess of non-degraded oligomer. The expanded repeat tracts in mRNA offer a number of binding sites for oligomers composed of complementary repeats, which may form a strong barrier for reverse transcription. Such misleading results can also be observed when the cells are transfected with unmodified single-stranded RNAs and when the repeat-targeting duplexes used at relatively high concentrations contain some excess of the single strand. This false silencing effect can be reversed by the removal of the short RNA fraction from the total RNA before reverse transcription (Additional file 5: Figure S4). In some cases, the removal of strongly bound oligomers may be difficult or even impossible to achieve. In such cases, the assessment of the total mRNA level can be performed by RT-PCR outside of the repeat region. However, the analysis of the protein level is necessary for an unbiased evaluation of the true silencing effects.

#### The evaluation of repeat targeting

For triplet-repeat-targeting strategies developed for polyQ diseases, the factor that enables allele and gene selectivity is a two- to three-fold difference in the length of complementary sequence in the normal and mutant allele [62]. In our study two different types of reagents composed of trinucleotide repeats were shown to be allele selective in *HTT *silencing: antisense oligomers (e.g., morpholino and PNA) and miRNA-like duplexes. The overall effect of the activities of these reagents is similar. The transcript level is not considerably decreased, whereas an efficient and selective downregulation of mutant protein is observed. In contrast, the mechanisms of activity for these two types of reagents are different. The antisense oligomers likely bind to the repeat tract without proteins, whereas the miRNA-like reagents interact with the complementary sequence within the RISC. In each case, the inhibition of mutant protein translation is achieved, which means that a translation blockade, e.g. for the 68 Q tract in huntingtin, is formed by either ~9 morpholino oligomers or ~3 CUG miRNA-loaded RISCs (according to the reported argonaute protein footprint on RNA [63]).

It is possible that the selectivity observed for RISC charged with miRNA-like acting siRNAs is achieved by cooperative action of silencing complexes, residing side by side on the repetitive sequence. This kind of synergistic effect was demonstrated for miRISC interaction with target sequence located within 3'UTR [64-66].

## Conclusions

Here we have presented and evaluated several oligonucleotide-based strategies which have been proposed for the treatment of polyQ diseases. The advantageous features of these strategies and their limitations are summarized in Table 1. It is clear that each strategy offers considerable advantages, and none of them should be disregarded at this point. These strategies require further development and testing to assess their potential therapeutic benefits and better understand their undesired side effects.

**Table 1:** A comparison of oligonucleotide-based strategies for polyQ diseases based on the desired properties of the reagents

PROPERTIES OF REAGENT					
STRATEGIES	target specificity	allele selectivity	universality	stability in cells	delivery in shRNA
**Specific silencing by RNAi**	uncertain	no	uncertain	uncertain	yes

**Replacement strategy**	uncertain	uncertain	uncertain	uncertain	yes

**SNP targeting**	uncertain	yes	no	uncertain	yes

**Repeat targeting by siRNA**	no	no	yes	uncertain	yes

**Repeat targeting by miRNA**	uncertain	yes	yes	uncertain	yes

**Repeat targeting by ASO**	uncertain	yes	yes	yes	no

For all the approaches specific gene silencing is the challenge and off-target effects should be carefully analyzed. The strategies can be designed to target the mutant allele only or both alleles. Allele-selective strategies were developed for SNP and repeat region-targeting. The repeat-targeting strategy is the most universal approach, and one carefully designed reagent could be used, in principle, for the treatment of most, if not all, polyQ diseases. Other strategies can be developed for a particular disease (nonallele-selective silencing, replacement strategy) or only for a group of patients suffering from a specific polyQ disorder (SNP-targeting). The clinically advantageous long-term effects of different reagents can be obtained either by the chemical modifications of synthetic reagents or by their cellular expression from suitable vectors.

Recent research has revealed that the pathogenesis of polyQ diseases is triggered not only by mutant protein but also by mutant transcript [67]. The exact contribution from a mutant transcript to the overall toxic effects, although unknown at present, needs to be taken into consideration when selecting and developing further the therapeutic approaches. The expression of toxic protein is lowered in each of the proposed strategies, but not all of them are effective in lowering the mutant transcript level. The normally expressed mutant transcript may be blocked from the pathogenic interactions with various protein factors by the same mechanism (reagent binding) which inhibits its translation [68]. On the other hand, transcript degradation and the resulting protein synthesis inhibition can be achieved by typical RNAi strategies. Some repeat-targeting miRNA-like reagents are capable of the specific inhibition of mutant protein translation, and some other trigger lowering of both, mutant transcript and protein (unpublished data). Thus, the rapidly developing technologies designed to inhibit mutant gene expression offer various options when applied to the treatment of polyQ diseases. These various gene silencing options may also be helpful in resolving the issue which has recently gained considerable importance: what are the shares of mutant transcript toxicity and mutant protein toxicity in the pathogenesis of polyQ diseases?

## Methods

### Cell culture and transfection

Fibroblasts from HD patients (GM04208, 21/44 CAG; GM04281, 17/68 CAG), SCA1 patients (GM06927, 29 CAG interrupted with 2 CAT/43 CAG) and SCA3 patients (GM06153, 18/69 CAG) (Coriell Cell Repositories) were grown in EMEM (Lonza) supplemented with 8% FBS (fetal bovine serum; Sigma-Aldrich), antibiotics (Sigma-Aldrich) and non-essential amino acids (Sigma-Aldrich). HeLa cells were grown in RPMI1640 medium (Lonza) supplemented with 10% FBS, antibiotics and vitamins (PAA).

The fibroblast cell transfections were performed using Lipofectamine 2000 (Life Technologies) according to the manufacturer's instructions. Briefly, the cells were plated in 25-cm^2 ^dishes 24 h prior to transfection at ~60% confluence. The transfection mixtures were prepared in EMEM with 5 μl of Lipofectamine, and the medium was changed 4 h after transfection. HeLa 229 cells LGC (Promochem, ATCC number: CCL-2.1) were transfected in 6-well plates with 100 nM siRNAs and 1 μg of plasmid per well using 2 μl of jetPRIME (Polyplus Transfection) according to the manufacturer's instructions. The efficiency of transfections was monitored using a BlockIT fluorescent siRNA (Life Technologies). The morpholino oligomers were diluted to 20 μM with 10 μM Endo-Porter (Gene Tools) in complete medium and added to the cells. Morpholino delivery was monitored using oligomer with 3' fluorescein. The PNA oligomers were diluted to 2 μM in EMEM and added to the cells with a medium exchange after 24 h.

All of the unmodified RNA oligonucleotides were synthesized by Metabion. The chemically modified oligomers were synthesized by Sigma-Aldrich, PANAGENE, Exiqon or Gene Tools. The sequences of the synthetic oligomers used in this study are presented in Figures. The siRNAs were annealed prior to transfection, by being combined in annealing buffer (Metabion) to a duplex concentration of 20 μM and incubated at 90°C for 1 minute and then at room temperature for 45 minutes. The formation of duplexes was confirmed by migration analysis (vs. single strands) in 2.5% agarose gel stained with GelRed (Biotium).

### RNA isolation and RT-PCR

Total RNA was isolated from cells using TRI Reagent (BioShop) according to the manufacturer's instructions. The RNA concentration was measured using a NanoDrop spectrophotometer. A total of 500 ng of RNA was reverse transcribed using Superscript II (Life Technologies) and random hexamer primers (Promega). The quality of the reverse transcription (RT) reaction was assessed using amplification of the GAPDH housekeeping gene. The primer sequences and their respective PCR products are listed in Additional file 2: Table S2. The reaction products were separated on 1.5% agarose gels in 0.5× TBE buffer and stained with ethidium bromide.

### Protein isolation and western blot

The cells were lysed in buffer containing 60 mM TRIS-base, 2% SDS, 10% sucrose and 2 mM PMSF. The protein concentration was estimated using a NanoDrop spectrophotometer. A total of 20 μg of protein was diluted in sample buffer containing 2-mercaptoethanol and boiled for 5 minutes. For ataxin-3 detection, the proteins were separated using Tris-HCl SDS-polyacrylamide gel electrophoresis (5% stacking/12% resolving gel) in Laemmli buffer. For huntingtin electrophoresis, a Tris-acetate SDS-polyacrylamide gel was used (5% stacking/4% resolving gel). After electrophoresis, the proteins were wet-transferred to a nitrocellulose membrane. All of the immunodetection steps were performed on an SNAPid (Millipore) in buffer containing 0.25% nonfat milk in PBS/0.1% Tween 20. For ataxin-3/GAPDH detection, the blots were probed with the primary antibodies anti-ataxin-3 (1:1000, MAB5360, Millipore) and anti-GAPDH (1:5000, MAB374, Millipore). The blots were then treated with a biotinylated secondary antibody (1:500, B7264, Sigma-Aldrich) and incubated with a streptavidin-AP (alkaline phosphatase) conjugate (1:2000, Millipore). The immunoreaction was detected using the BCIP/NBT substrate (Sigma-Aldrich). For huntingtin and plectin detection, the blots were probed with the primary antibodies: anti-huntingtin (1:1000, MAB2166, Millipore) and anti-plectin (1:1000, ab83497, Abcam), and then with HRP-conjugated secondary antibodies (1:500, A9917, Sigma-Aldrich; 711035152, Jackson ImmunoResearch). The immunoreaction was detected using the ChemiFast Chemiluminescent Substrate (SYNGENE).

### The construction of the ATXN3 plasmid

The cDNA sequence of ataxin-3 (~1.8 kb) containing 8 CAG repeats was cloned into a pGEM-T Easy vector (Promega). Site-directed mutagenesis of an insert was performed with primers MUT_R and MUT_F (sequences given in Additional file 2: Table S2) using a Phusion Kit (Finnzymes). The mutated ATXN3 insert was then cloned into pIRES2-AcGFP1 (Clontech) using NheI and SacI restriction enzymes (Fermentas).

### Statistical analysis

All experiments were repeated at least three times. The statistical significance of silencing was assessed with a one-sample *t*-test with the hypothetical value of 1 assigned to cells treated with control siRNA (C). Selected data were compared using an unpaired *t*-test. Two-tailed *P *values of less than 0.05 were considered significant.

## Abbreviations

ASO: Antisense oligonucleotide; HD: Huntington's disease; LNA: Locked nucleic acid; miRNA: microRNA; polyQ: Polyglutamine; PNA: Peptide nucleic acid; RISC: RNA-induced silencing complex; RNAi: RNA interference; SCA: Spinocerebellar ataxia; shRNA: Short hairpin RNA; siRNA: Short interfering RNA; SNP: Single-nucleotide polymorphism.

## Competing interests

The authors declare that they have no competing interests.

## Authors' contributions

WJK conceived and coordinated the study. The experiments were designed and performed by AF (specific silencing in HD, SNP-targeting in SCA1, repeat-targeting), MO (SNP-targeting in HD), PMS (contribution to repeat-targeting), JPW (gene replacement strategy), JW-K (SNP-targeting in SCA3), and AM (contribution to repeat-targeting). AF analyzed the data and prepared the figures. WJK and AF wrote the paper. All authors read and approved the final manuscript.

## Supplementary Material

Additional file 1 **Figure S1**. The activity of siRNAs targeting both alleles of *HTT*. (A) The target sites and nucleotide sequences of siRNAs directed at the mRNA of the *HTT *gene (lowercase for RNA, uppercase for DNA). (B) The RT-PCR analysis of *HTT *expression at the transcript level in the HD fibroblasts (GM04281) transfected with 50 nM siRNA HD1 at the indicated time points. (C) The western blot analysis of the huntingtin levels for the experiment described in (B). (D) The RT-PCR analysis of the HTT transcript levels in the HD fibroblasts (GM04281) at 72 h after transfection with 50 nM siRNAs HD1, HD2 and HD3. (E) The western blot analysis of the huntingtin levels in the HD fibroblasts (GM04281) at 72 h after transfection with 2, 10 or 50 nM siRNA HD1. C - the reference bar indicates the *HTT *expression level in the cells transfected with control siRNA. In the graphs, the signal intensities were normalized to GAPDH mRNA or protein levels and compared using a one-sample *t*-test. The error bars represent standard deviations. The *P*-value is indicated by asterisks (* p < 0.001; ** 0.001 < p < 0.05). Click here for file

Additional file 2 **Supplementary methods and supplementary tables**. Click here for file

Additional file 3 **Figure S2**. The activity of siRNAs targeting SNP sites in the HD model. (A) The target sites and nucleotide sequences of the siRNAs directed at rs1065745 SNP in the *HTT *gene. The nucleotides in the antisense strand that directly interact at the SNP site and additional mismatches with the target sequence are marked in bold. The mismatches introduced into the siRNA duplex are in gray boxes. RNA is in lowercase; DNA is in uppercase. (B) The RT-PCR analysis of the *HTT *expression at the transcript level in the HD fibroblasts (GM04208) at 72 h after transfection with 50 nM siRNAs. (C) The RT-PCR analysis of the HTT transcript level in the HD fibroblasts at 72 h after transfection with 2, 10, 50 or 200 nM siRNA G16. (D) The western blot analysis for the experiment described in (C). C - the reference bar indicates the *HTT *expression levels in the cells transfected with control siRNA. In the graphs, the signal intensities were normalized to GAPDH mRNA level and compared using a one-sample *t*-test. The error bars represent the standard deviations. The *P*-value is indicated by asterisks (* p < 0.001; ** 0.001 < p < 0.05). Click here for file

Additional file 4 **Figure S3**. The activity of siRNAs targeting SNP sites in the SCA1 model. (A) The target sites and the nucleotide sequences of siRNAs directed at the rs179990 SNP in the *ATXN1 *gene. The nucleotides in the antisense strand that directly interact at the SNP site are marked in bold. The mismatches introduced into the siRNA duplex are in gray boxes. RNA is in lowercase; DNA is in uppercase. (B) The RT-PCR analysis of *ATXN1 *expression at the transcript level in the SCA1 fibroblasts (GM06927) at 72 h after transfection with 50 nM siRNAs. (C) The RT-PCR analysis of ATXN1 transcript level in the SCA1 fibroblasts at 72 h after transfection with 2, 10, 50 or 200 nM siRNA G10. C - the reference bar indicates the *ATXN1 *expression level in the cells transfected with the control siRNA. In the graphs, the signal intensities were normalized to the GAPDH mRNA level and compared using a one-sample *t*-test. The error bars represent standard deviations. The *P*-value is indicated by asterisks (* p < 0.001; ** 0.001 < p < 0.05). Click here for file

Additional file 5 **Figure S4**. Difficulties in the RT-PCR including the amplification of the expanded repeat tract. The RT-PCR analysis of the *HTT *alleles' transcript levels in the HD fibroblasts (GM04281) at 24 h after transfection with 10 nM control siRNA (C) or RNA (CUG)7. The two lanes on the left represent RT-PCR performed in standard conditions. The two lanes on the right include RNA purification from the fraction of short RNAs before RT-PCR. Click here for file
