# Supplementary figures and images for: An evaluation of oligonucleotide-based therapeutic strategies for polyQ diseases
Source: BMC Mol Biol. 2012 Mar 7;13:6. doi: 10.1186/1471-2199-13-6 (PMC3359213; doi:10.1186/1471-2199-13-6)

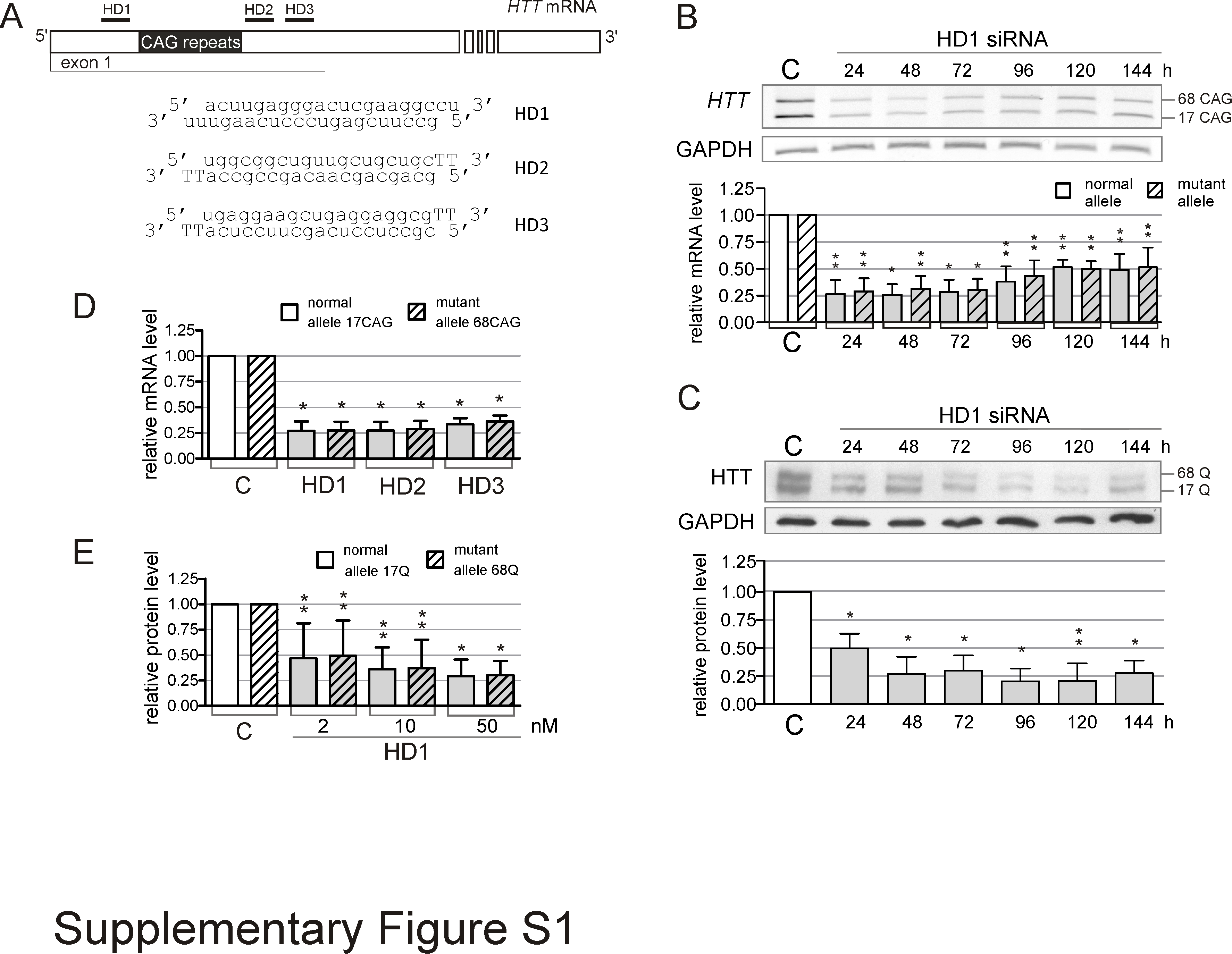

Supplement: Additional file 1 — Figure S1. The activity of siRNAs targeting both alleles of HTT. (A) The target sites and nucleotide sequences of siRNAs directed at the mRNA of the HTT gene (lowercase for RNA, uppercase for DNA). (B) The RT-PCR analysis of HTT expression at the transcript level in the HD fibroblasts (GM04281) transfected with 50 nM siRNA HD1 at the indicated time points. (C) The western blot analysis of the huntingtin levels for the experiment described in (B). (D) The RT-PCR analysis of the HTT transcript levels in the HD fibroblasts (GM04281) at 72 h after transfection with 50 nM siRNAs HD1, HD2 and HD3. (E) The western blot analysis of the huntingtin levels in the HD fibroblasts (GM04281) at 72 h after transfection with 2, 10 or 50 nM siRNA HD1. C - the reference bar indicates the HTT expression level in the cells transfected with control siRNA. In the graphs, the signal intensities were normalized to GAPDH mRNA or protein levels and compared using a one-sample t-test. The error bars represent standard deviations. The P-value is indicated by asterisks (* p < 0.001; ** 0.001 < p < 0.05). [file 1471-2199-13-6-S1.TIFF]

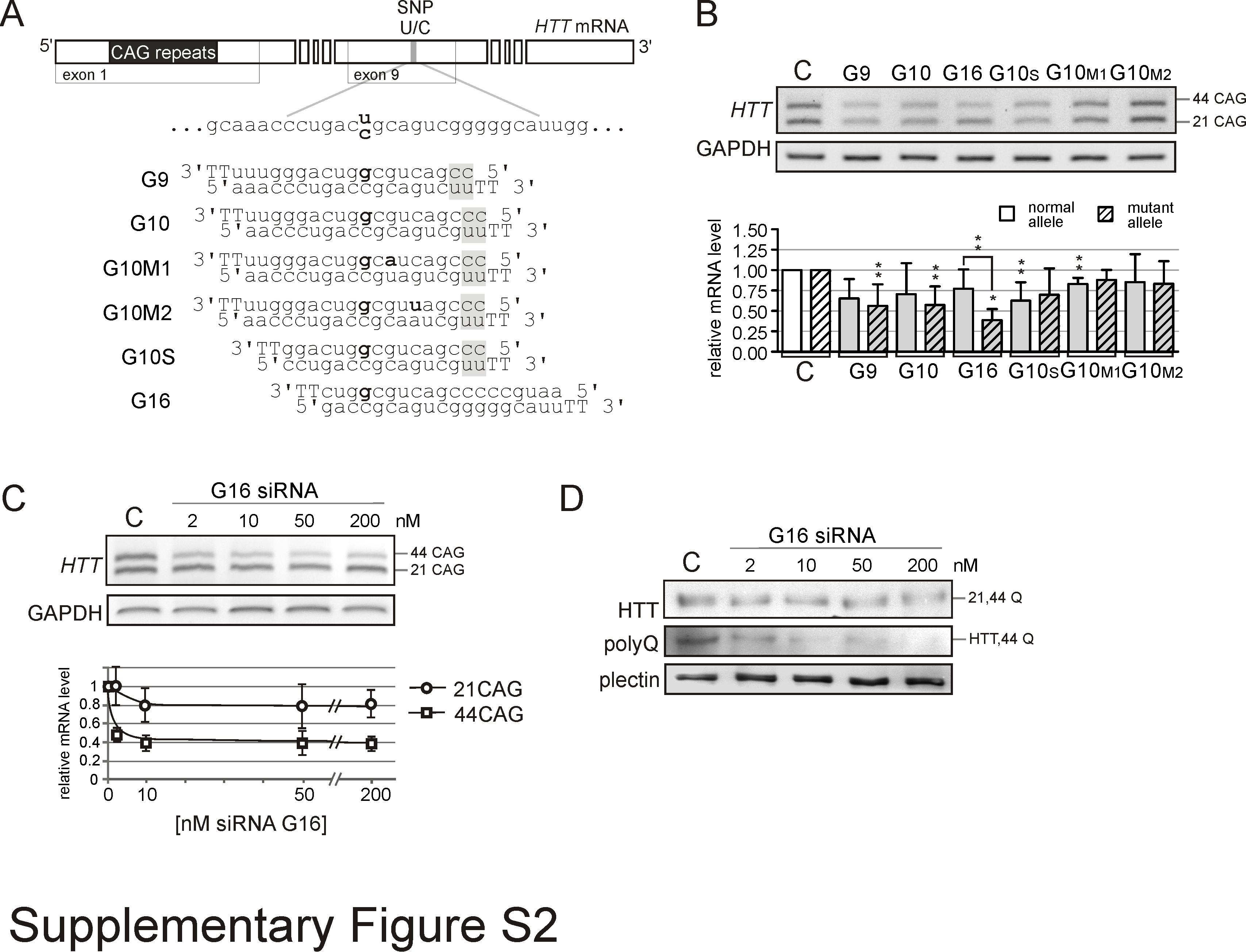

Supplement: Additional file 3 — Figure S2. The activity of siRNAs targeting SNP sites in the HD model. (A) The target sites and nucleotide sequences of the siRNAs directed at rs1065745 SNP in the HTT gene. The nucleotides in the antisense strand that directly interact at the SNP site and additional mismatches with the target sequence are marked in bold. The mismatches introduced into the siRNA duplex are in gray boxes. RNA is in lowercase; DNA is in uppercase. (B) The RT-PCR analysis of the HTT expression at the transcript level in the HD fibroblasts (GM04208) at 72 h after transfection with 50 nM siRNAs. (C) The RT-PCR analysis of the HTT transcript level in the HD fibroblasts at 72 h after transfection with 2, 10, 50 or 200 nM siRNA G16. (D) The western blot analysis for the experiment described in (C). C - the reference bar indicates the HTT expression levels in the cells transfected with control siRNA. In the graphs, the signal intensities were normalized to GAPDH mRNA level and compared using a one-sample t-test. The error bars represent the standard deviations. The P-value is indicated by asterisks (* p < 0.001; ** 0.001 < p < 0.05). [file 1471-2199-13-6-S3.TIFF]

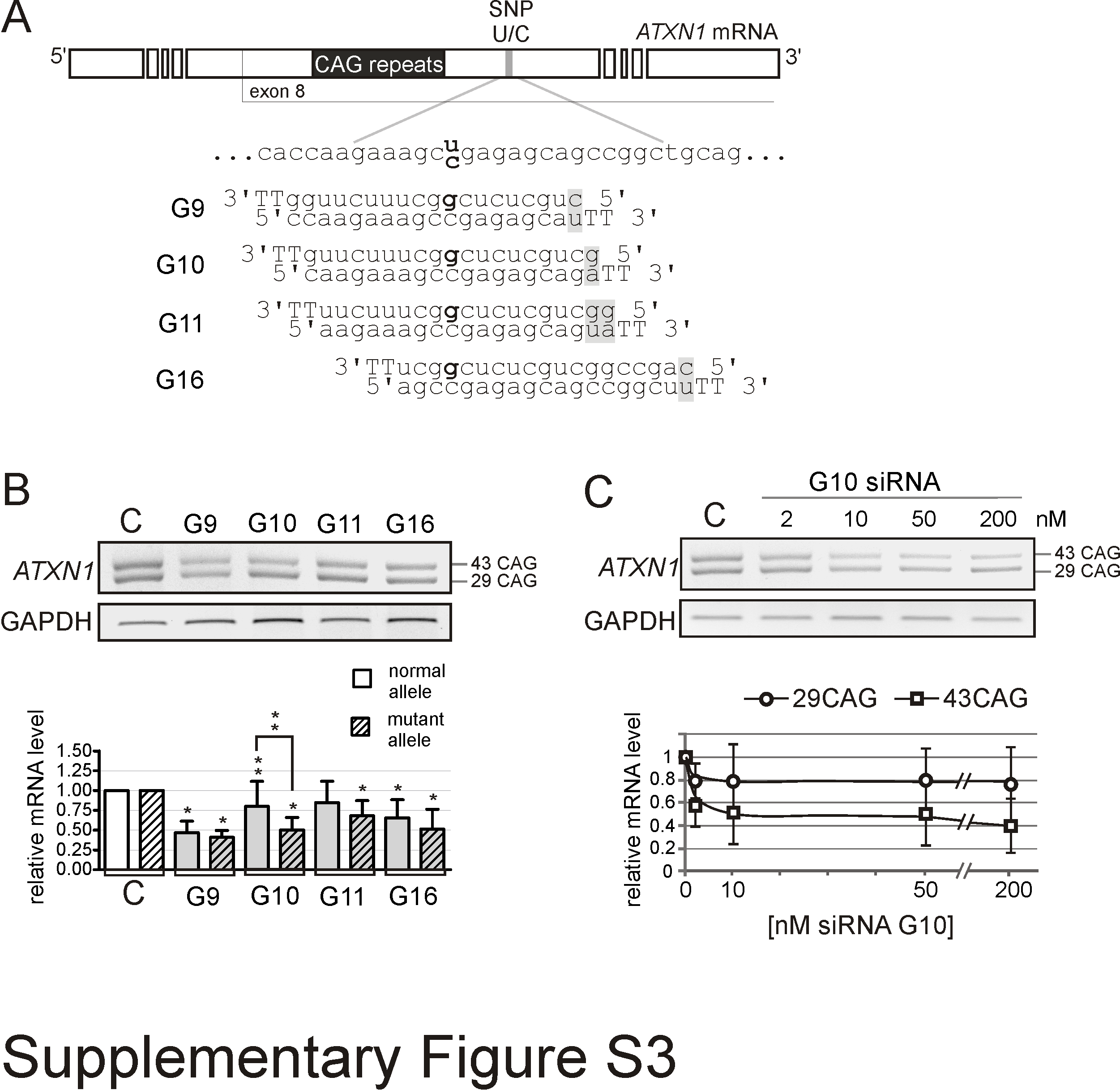

Supplement: Additional file 4 — Figure S3. The activity of siRNAs targeting SNP sites in the SCA1 model. (A) The target sites and the nucleotide sequences of siRNAs directed at the rs179990 SNP in the ATXN1 gene. The nucleotides in the antisense strand that directly interact at the SNP site are marked in bold. The mismatches introduced into the siRNA duplex are in gray boxes. RNA is in lowercase; DNA is in uppercase. (B) The RT-PCR analysis of ATXN1 expression at the transcript level in the SCA1 fibroblasts (GM06927) at 72 h after transfection with 50 nM siRNAs. (C) The RT-PCR analysis of ATXN1 transcript level in the SCA1 fibroblasts at 72 h after transfection with 2, 10, 50 or 200 nM siRNA G10. C - the reference bar indicates the ATXN1 expression level in the cells transfected with the control siRNA. In the graphs, the signal intensities were normalized to the GAPDH mRNA level and compared using a one-sample t-test. The error bars represent standard deviations. The P-value is indicated by asterisks (* p < 0.001; ** 0.001 < p < 0.05). [file 1471-2199-13-6-S4.TIFF]

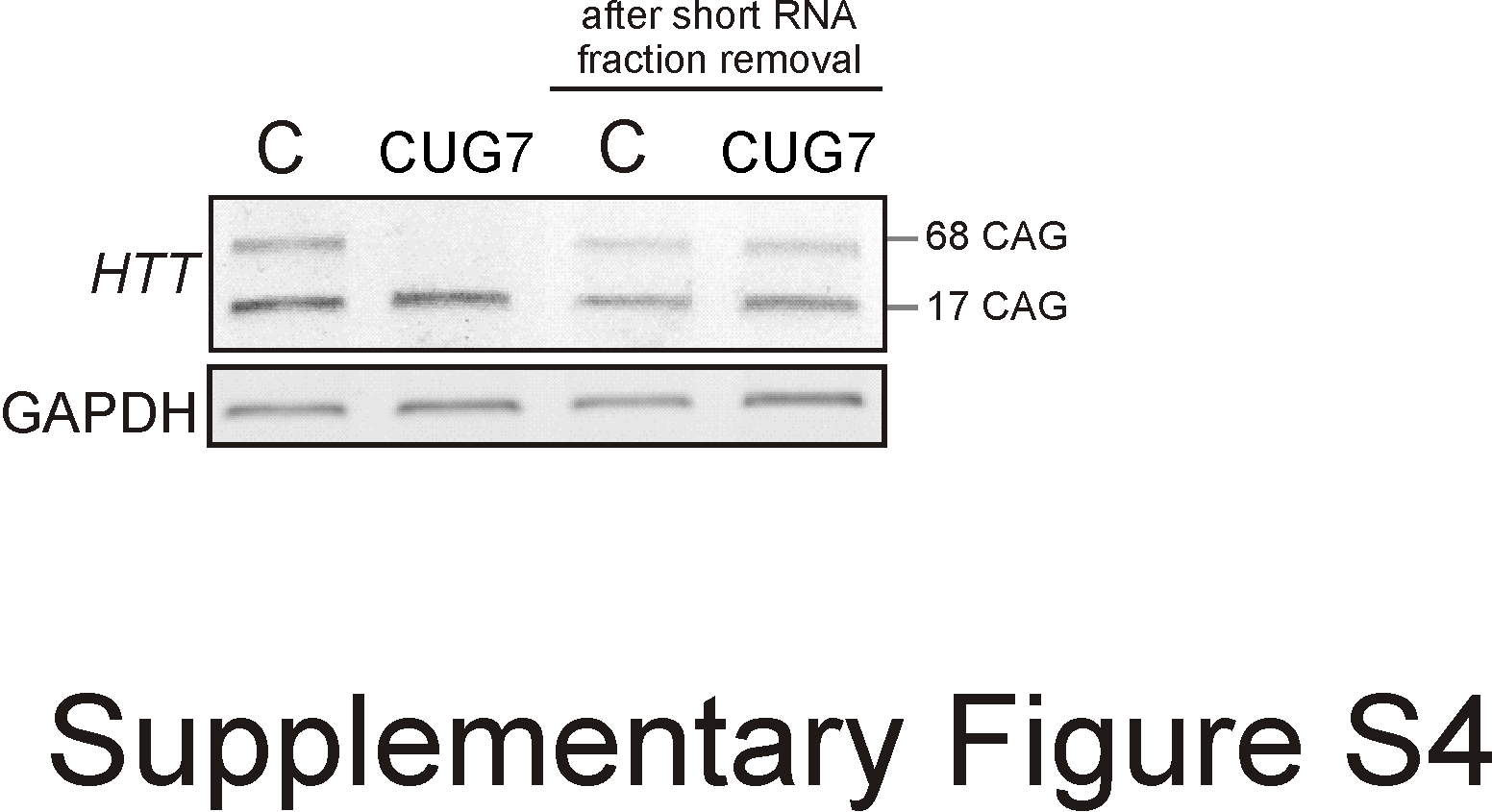

Supplement: Additional file 5 — Figure S4. Difficulties in the RT-PCR including the amplification of the expanded repeat tract. The RT-PCR analysis of the HTT alleles' transcript levels in the HD fibroblasts (GM04281) at 24 h after transfection with 10 nM control siRNA (C) or RNA (CUG)7. The two lanes on the left represent RT-PCR performed in standard conditions. The two lanes on the right include RNA purification from the fraction of short RNAs before RT-PCR. [file 1471-2199-13-6-S5.TIFF]
